# Diet for Schoolboys

**Published:** 1896-08-01

**Authors:** 


### Diet for Schoolboys.

II.—THE QUESTION OF MEALS AND FOOD.

The question of meals and individual articles of food has many important bearings. If we allow six hours before a full meal leaves the stomach, and two hours for a light one, we find considerable difficulty in arranging the hours so that there may be a continuous supply of nutrient material in the duodenum during the waking hours, and during these only; but in children the process is more rapid, so that four hours should be about the maximum interval. Domestic reasons in this country usually prevent breakfast being earlier than half-past eight, dinner comes at half-past one, and the lighter evening meal about six or seven for most children. Now these intervals are clearly too long.

The agricultural labourer supplements them with three snacks or luncheons—in the early morning, at eleven, and four. Something of the same kind must be attempted for the schoolboy. Even if we take the view that an hour or two of rest is good for the stomach before meals, the schoolboy refuses to carry out our plan, and stops his gnawing appetite by indigestible delicacies. First, then, as to the time before breakfast when there is work to be done. Here there is general agreement that for all but the strongest boys some light refreshment is desirable, if for no other reason than that it prevents a large number of colds, and indeed, infectious disorders, since everyone is far more liable to these affections when the system is depressed. Milk, cocoa, or a biscuit will suffice, and care must be taken to see that the meal is not evaded by late risers and by those boys who fear the reproach of delicacy. Milk should always be first boiled in hot water from its tendency to be infected with disease germs; cocoa is a mild, safe food, containing a little fat and starch, and preferable to tea and coffee for growing children. Indeed, without accepting the common view of the dangers of tea, neither tea, coffee, alcohol, nor tobacco is desirable for the rapidly-growing organism, for they probably hinder metabolism by shielding certain tissues from destruction. French children often have a cup of thin soup at this hour, and the custom might well be adopted here, at least as a change from the round of cocoa and milk. If water is preferred for drinking with biscuits, too much care cannot be taken to ensure its purity. It is, like milk, easily contaminated, and by no means easy to purify when once infected. Like milk, it is safer if boiled, but the taste of boiled water is so unsatisfactory that this plan can hardly be insisted on. The ordinary filters are worse than useless, merely collecting numbers of germs which actually multiply within them, and letting through some of the most dangerous. The breakfast proper should be made a substantial meal, not a mere bread and butter preparation for dinner. Besides plenty of these articles we must add porridge, eggs, fish, bacon, pork, or some such nitrogenous food. Tea or coffee can hardly be avoided, but plenty of milk, butter, and sugar must be given and even insisted on. Cleanliness and good cooking are all essential, and sufficient time for slow eaters and care that the bigger boys do not consume more than their share of the more delicate morsels at all meals is essential. The long interval till one or half-past one, when dinner can be looked for, must be abridged by a cup of milk, a bun, or a piece of bread and cheese towards noon. The main food supply must be given at breakfast and dinner, when the digestion is active and the body untired, so that food may be provided ready for the time of work and digested before sleep. At dinner the chief supply of meat has to be consumed, vegetables eaten, and a full meal taken by every boy in health. If anyone fails in this the cause must be inquired into. Some, of course, are difficult to please on any diet, but most boys, if they can be kept from the pastry shop till after meals, and have a fair variety of well-cooked food, are content.

The food, as Dr. Dukes remarks, may be deficient in quantity while plenty is provided if the weaker boys are badly served or the meals are too few, since few boys can eat all they need at two or three meals, or if the diet be too monotonous, or if it is bolted in haste. Besides this the quality of the meat may be bad, the cooking defective, the potatoes and other vegetables served in a state to cause disgust, and the puddings heavy and indigestible. The inability of the Germanic races to cook vegetables in an appetising form leads to their eating every kind together with their meat, for the sake of the flavour. This leaves less opportunity for bread, of which we seem to consume comparatively little. The defect is to some extent made up by the number and variety of the puddings when these are well cooked. Hence the value of good rice, treacle, and fruit puddings. Another point on which Dr. Dukes has insisted is, that if an economical dietary has to be chosen, and the meat supply kept moderate, cheese and the vegetable albumens from oatmeal, lentils, and peas must be increased, though these are less easy of digestion. The evening meal need not include nitrogenous food, but may be made a substantial one if we give plenty of sugar in the form of jam, marmalade, and treacle, as well as butter and milk. If this is taken not later than six o'clock, the weaker boys will be better for milk or a bun before bed time, but anything like a supper should be carefully avoided.

Variety does not imply a constant supply of a number of different articles together. Indeed, the more delicate digestions succeed best on meals of one or two kinds of foodstuff, provided these are changed from day to day. Change of the menu from day to day and, in fact, from week to week is of the greatest importance. Little has been said about alcohol, because young and healthy persons, properly fed with appetising food, are better without it. Still, there is some truth in Savory's remark that a little beer served at dinner, while modern customs exist, prevents boys seeking it surreptitiously, since they generally want what is not allowed. Every effort is required to afford a wholesome and palatable diet, to satisfy every healthy craving of the stomach, and to ensure that the food chosen is furnished at suitable times and in such a way that every boy has ample leisure to consume it.

